# *In Utero* Cigarette Smoke Affects Allergic Airway Disease But Does Not Alter the Lung Methylome

**DOI:** 10.1371/journal.pone.0144087

**Published:** 2015-12-07

**Authors:** Kenneth R. Eyring, Brent S. Pedersen, Ivana V. Yang, David A. Schwartz

**Affiliations:** 1 Department of Medicine, School of Medicine, University of Colorado, Aurora, Colorado, United States of America; 2 Department of Immunology, School of Medicine, University of Colorado, Aurora, Colorado, United States of America; National Jewish Health, UNITED STATES

## Abstract

Prenatal and postnatal cigarette smoke exposure enhances the risk of developing asthma. Despite this as well as other smoking related risks, 11% of women still smoke during pregnancy. We hypothesized that cigarette smoke exposure during prenatal development generates long lasting differential methylation altering transcriptional activity that correlates with disease. In a house dust mite (HDM) model of allergic airway disease, we measured airway hyperresponsiveness (AHR) and airway inflammation between mice exposed prenatally to cigarette smoke (CS) or filtered air (FA). DNA methylation and gene expression were then measured in lung tissue. We demonstrate that HDM-treated CS mice develop a more severe allergic airway disease compared to HDM-treated FA mice including increased AHR and airway inflammation. While DNA methylation changes between the two HDM-treated groups failed to reach genome-wide significance, 99 DMRs had an uncorrected p-value < 0.001. 6 of these 99 DMRs were selected for validation, based on the immune function of adjacent genes, and only 2 of the 6 DMRs confirmed the bisulfite sequencing data. Additionally, genes near these 6 DMRs (*Lif*, *Il27ra*, *Tle4*, *Ptk7*, *Nfatc2*, and *Runx3*) are differentially expressed between HDM-treated CS mice and HDM-treated FA mice. Our findings confirm that prenatal exposure to cigarette smoke is sufficient to modify allergic airway disease; however, it is unlikely that specific methylation changes account for the exposure-response relationship. These findings highlight the important role in utero cigarette smoke exposure plays in the development of allergic airway disease.

## Introduction

Smoking during pregnancy has long been identified as an independent risk factor for short term maternal and fetal outcomes, such as intrauterine growth restriction, ectopic pregnancy, premature birth, and developmental deficiencies. Furthermore, this exposure can lead to long-lasting changes in disease susceptibility, including asthma, behavioral disorders, obesity, and respiratory illness [1]. In fact, pre- and postnatal cigarette smoke exposure is one of the strongest environmental risk factors of asthma [2], and the risk of developing asthma symptoms is doubled when exposed early in life [3]. However, the mechanisms that result in childhood asthma following maternal smoking during pregnancy are largely unknown.

Despite the well know risks of smoking during pregnancy, an estimated 23% of women reported they smoked 3 months prior to pregnancy while 11% of pregnant women continue to smoke throughout pregnancy in the United States [4]. In the same report, 16% of women smoked 4 months after pregnancy. However, this may underestimate those affected by cigarette smoke as it does not include second hand or environmental cigarette smoke.

Cigarette smoke is known to influence epigenetic mechanisms, including epigenetic machinery [5, 6], post-translational histone modifications [7, 8], and DNA methylation [9, 10]. DNA methylation changes due to cigarette smoke can occur quite rapidly and persist for extended periods of time [11, 12]. One known association is the methylation of RUNX3 with a history of smoking in bladder tumors [13]. We have previously reported that Runx3 is differentially methylated in allergic airway disease in mice [14]; this is supported by the report that Runx3 deficient mouse spontaneously develop a phenotype resembling allergic airway disease [15].

Based on these observations, we hypothesized that cigarette smoke exposure during prenatal development generates long lasting differential methylation altering transcriptional activity that corresponds with altered disease.

## Materials and Methods

### Mice

C57BL/6J mice were purchased from Jackson Laboratories. Animals were housed under standard conditions and protocols were approved by the Institutional Animal Care and Use Committee of the University of Colorado Denver.

### Cigarette Smoke Exposure

12 week old C57BL/6J females were exposed to cigarette smoke at ~50 mg/m^3^ TSP (equivalent to about a pack a day or a heavy smoker) or filtered air for 5 hours/day, 5 days/week for 4 weeks (1 week acclimation included) prior to mating with C57BL/6J males. Cigarette smoke was generated by the TE-10 smoking machine (Teague Enterprises) from 2R4F research cigarettes (University of Kentucky). Cigarette smoke contained a mixture of both side and mainstream smoke. Exposure was continued until birth of pups at which time all exposures were stopped and mothers and pups were placed under normal housing conditions.

### Phenotyping

Allergic airway disease was induced using an adapted house dust mite model [16]. Briefly, mice were sensitized to 10 μg of filtered house dust mite extract (HDM, GREER Labs) or saline through intraperitoneal (i.p) injection on days 0 and 7 followed by sensitization on days 14 and 15 with 5 μg HDM or saline administered intratracheally using a microsprayer (Penn Century). On day 17, mice were anesthetized by an i.p. injection of pentobarbital sodium (60 mg/kg). Following tracheostomy, pancuronium bromide (0.25 mg/kg) was administered, and mice were ventilated on a small animal ventilator (flexiVent FV-FXM1; SCIREQ). Airway resistance was measured through forced oscillation techniques (flexiVent FV-FXM1; SCIREQ) over increasing doses of methacholine. Following procedure, a cardiac stick was performed to euthanize the mouse and collect blood, then whole lung lavage (WLL) was collected. Lung tissue was perfused with phosphate buffered saline then snap frozen in liquid nitrogen. Cytokines in the lung lavage and IgE in the serum were measured using ELISA MAX Standard Sets and protocols from BioLegend. Additional information is provided in the supplemental methods (S1 Appendix).

### Bisulfite Sequencing and Data Analysis

To measure DNA methylation in whole lung tissue, bisulfite sequencing was performed utilizing Agilent’s SureSelect Methyl-Seq Target Enrichment System for Illumina Multiplexed Sequencing. Experimental procedures followed SureSelect Human Methyl-Seq Protocol Version B using SureSelect Methyl-Seq Reagent Kit and Mouse Methyl-Seq Capture Library. Additional information is provided in the supplemental methods (S1 Appendix).

Bisulfite-sequencing reads were handled using bwa-meth [17] which also tabulated percent methylation at each CpG motif. Correlating sets of adjacent CpG sites were clustered together using the Adjacent Site Clustering algorithm [18]. Each cluster was required to have a minimum of three CpG sites to constitute a cluster. Methylation clusters were analyzed using a beta regression weighted on sequence read depth, and multiple testing correction was performed using the Benjamini-Hocheberg method [19].

### Pyrosequencing

DMRs identified through bisulfite sequencing were confirmed through pyrosequencing PCR on Qiagen’s Pyromark MD. Additional information is provided in the supplemental methods (S1 Appendix).

### RT-PCR

Differential expression was tested through qRT-PCR on the Viia7 Real-Time PCR system (Applied Biosystems) using Taqman assays (Applied Biosystems). Additional information is provided in the supplemental methods (S1 Appendix).

### Statistics

Data were expressed as mean ±SEM. Individual comparisons between groups were confirmed by a 1-tailed Mann-Whitney U test because we were testing only 1 outcome, that *in utero* cigarette smoke caused increased allergic airway inflammation. Significant differences between groups were identified by analysis of variance. For validation, pyrosequencing data was analyzed using a 1 tailed Mann-Whitney U test for HDM-treated CS mice versus HDM-treated FA mice. A 2 tailed Mann-Whitney U test was applied to qRT-PCR data to compare HDM-treated CS mice versus HDM-treated FA mice. GraphPad Prism version 5.04 (GraphPad Software, La Jolla, CA) was used to perform statistical calculations. Pathway analysis was performed using Ingenuity Pathway Analysis (IPA) software.

## Results

### In utero cigarette smoke exposure alters HDM-induced allergic airway disease

**Fig 1:**
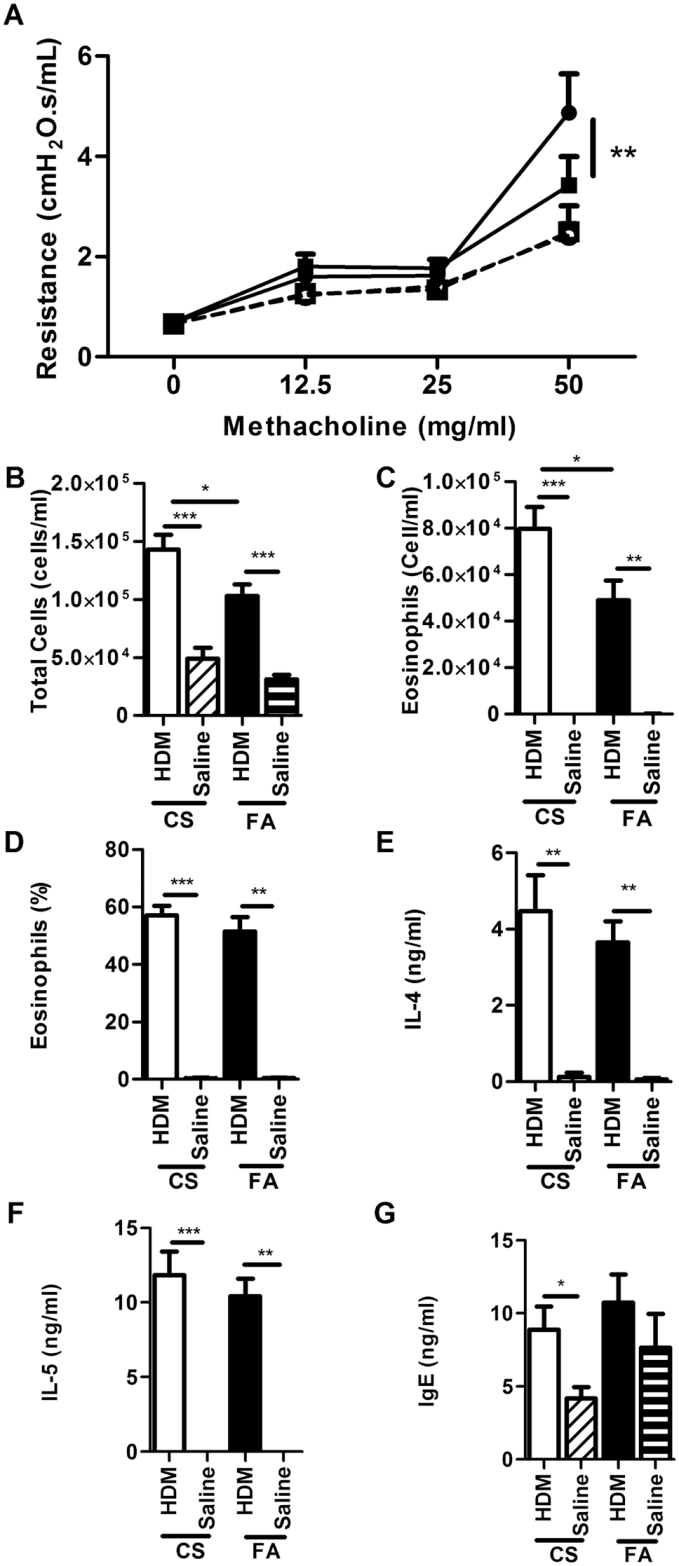
In utero cigarette smoke exposure enhanced the severity of HDM-induced allergic airway disease. (A) airway hyperresponsiveness (HDM-treated FA solid line closed square, HDM-treated CS solid line closed circle, saline-treated FA dashed line open square, and saline-treated CS dashed line open circle), (B) total cell in WLL, and (C) concentration of eosinophils, (D) percentage of eosinophils in the WLL, (E) WLL IL-4 cytokine levels, (F) WLL IL-5 cytokine levels, and (G) total IgE levels in the serum (HDM-treated CS white bar, HDM-treated FA black bar, saline-treated CS white bar with black dots, and saline-treated FA black bar with white dots, * p-value < 0.05, ** p-value < 0.01, *** p-value < 0.001).

These results support previously published data that mice exposed during gestation to cigarette smoke (CS) develop a more severe allergic airway disease phenotype [20–24]. HMD-treated mice exposed to CS during gestation demonstrate increased AHR compared to HDM-treated mice exposed to filtered air (FA) during gestation. Following challenge with HDM, enhanced airway inflammation is observed in CS mice compared to FA mice with an increase in total cells and eosinophils in WLL (Fig 1A–1D). Both HDM-treated CS and FA mice differ from their respective saline-treated mice counterparts in terms of AHR and cellular infiltration. There is no statistical difference observed for saline-treated mice treated with cigarette smoke (Fig 1A–1D).

We observed no differences in IL-4 and IL-5 concentrations in WLL between HDM-treated CS and FA mice (Fig 1E–1G). HDM-treatment resulted in increased concentrations of IL-4 and IL-5 in WLL compared to saline-treatment in both CS and FA treated mice. However, only HDM-treated CS mice demonstrated a significant increase in total IgE in the serum compared to saline-treated CS mice, and the lack of differences between HDM-treated and saline-treated mice is limited by the measurement of total IgE and not antigen specific IgE (Fig 1E–1G).

### DNA methylation changes due to prenatal smoke exposure in allergic airway disease

**Fig 2:**
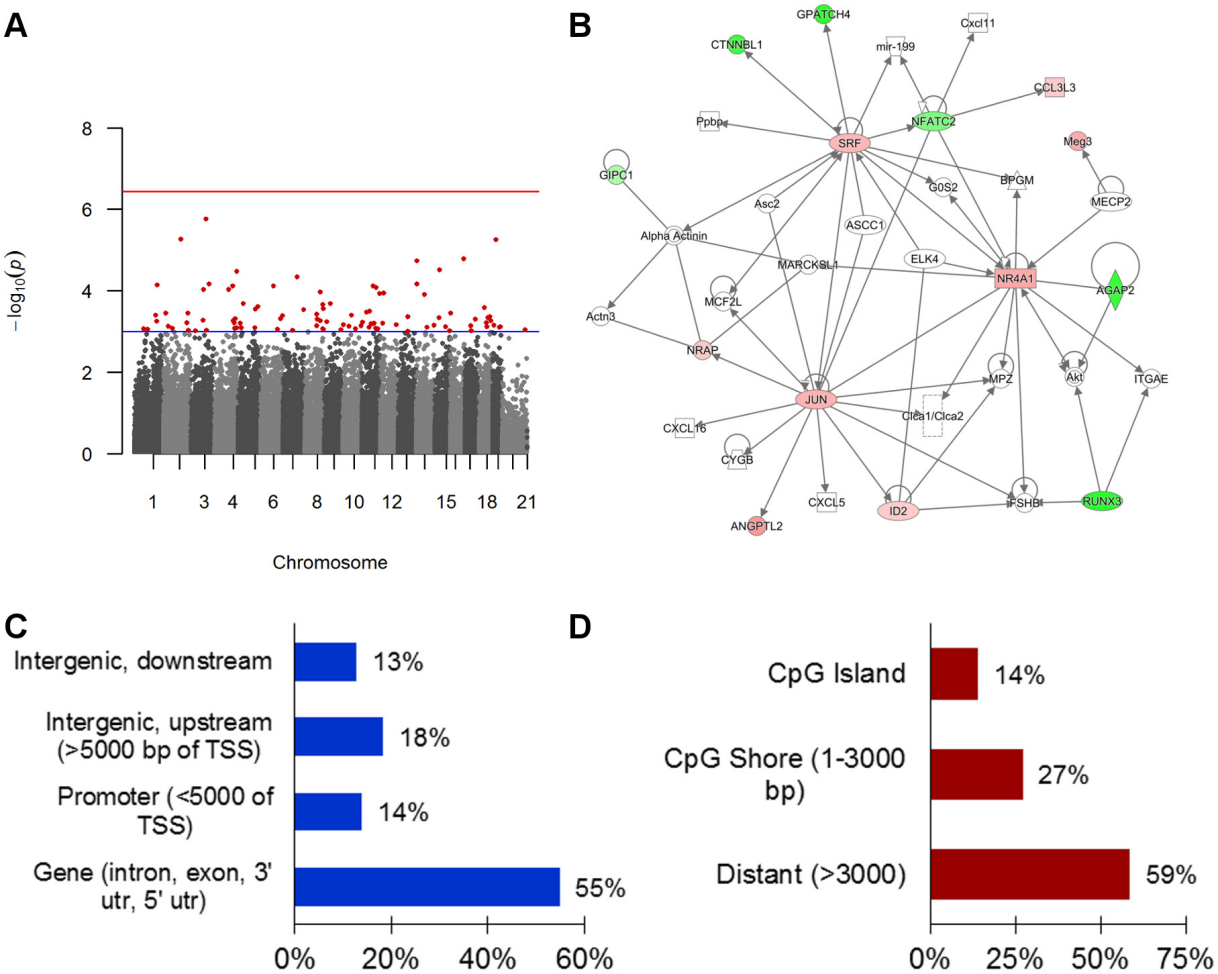
DMRs lack genome-wide association with *in utero* cigarette smoke exposure in allergic airway disease. (A) Manhattan plot of the p-values from a weighted beta regression for HDM-treated CS vs. HDM-treated FA mice. Each dot represents a p-value for correlating CpG clusters as identified through A-clustering with a minimum of 3 adjacent CpGs within a cluster. The red horizontal line is the estimated genome-wide significance threshold of p = 3.6 x 10^−7^; the blue line is the threshold for suggestive association (p = 0.001). Red dots denote suggestive DMRs with an unadjusted p-value < 0.001 (n = 99). (B) A molecular network identified by Ingenuity Pathway Analysis of the genes nearest to the 99 DMRs. This network demonstrates that these genes have a number of direct and shared interactions with each other. Network analysis was performed using only direct interactions and a minimum network score of 20. Legend: genes are colored red (hypermethylated) or green (hypomethylated), horizontal ellipse = transcriptional regulator, square = cytokine, double circle = group/complex, vertical diamond = enzyme, vertical rectangle = G-protein coupled receptor, circle = other, and triangle = phosphatase. Genomic distribution of the top 99 DMRs by relationship to (C) gene and (D) CpG Island.

Agilent’s SureSelect targeted methyl-sequencing was performed in whole lung tissue to determine methylation patterns at specific sites throughout the genome. On average, each sample approximated 64 million reads with 88% of the reads falling within the targeted regions with greater than 50% of the regions with 20x coverage (S1 Table). Comparing DNA methylation between the HDM-treated groups failed to identify significant DMRs after multiple testing correction. Due to the lack of association and the increased noise in the data due to the admixture of cell types in whole lung tissue, we therefore reduced the statistical threshold to investigate suggestive DMRs. There are 99 suggestive DMRs with an uncorrected p-value < 0.001 (Fig 2A and S2 Table). These DMRs have an average length of 93 base pairs, are primarily found in gene bodies (intron, exon, 3’ untranslated region, or 5’ untranslated region; n = 56 or 57%; Fig 2C), and areas outside of CpG islands and shores (>3000 bases from the island; n = 58 or 59%; Fig 2D). 45 DMRs are hypomethylated.

DMRs are annotated based on nearest gene, and these genes were uploaded into IPA. Despite the small number of genes entered, 65 canonical pathways are enriched of which many involved immune function (S3 Table). Ingenuity network analysis on the 99 DMRs identified 6 networks with a minimum score > 20 (Fig 2B).

**Fig 3:**
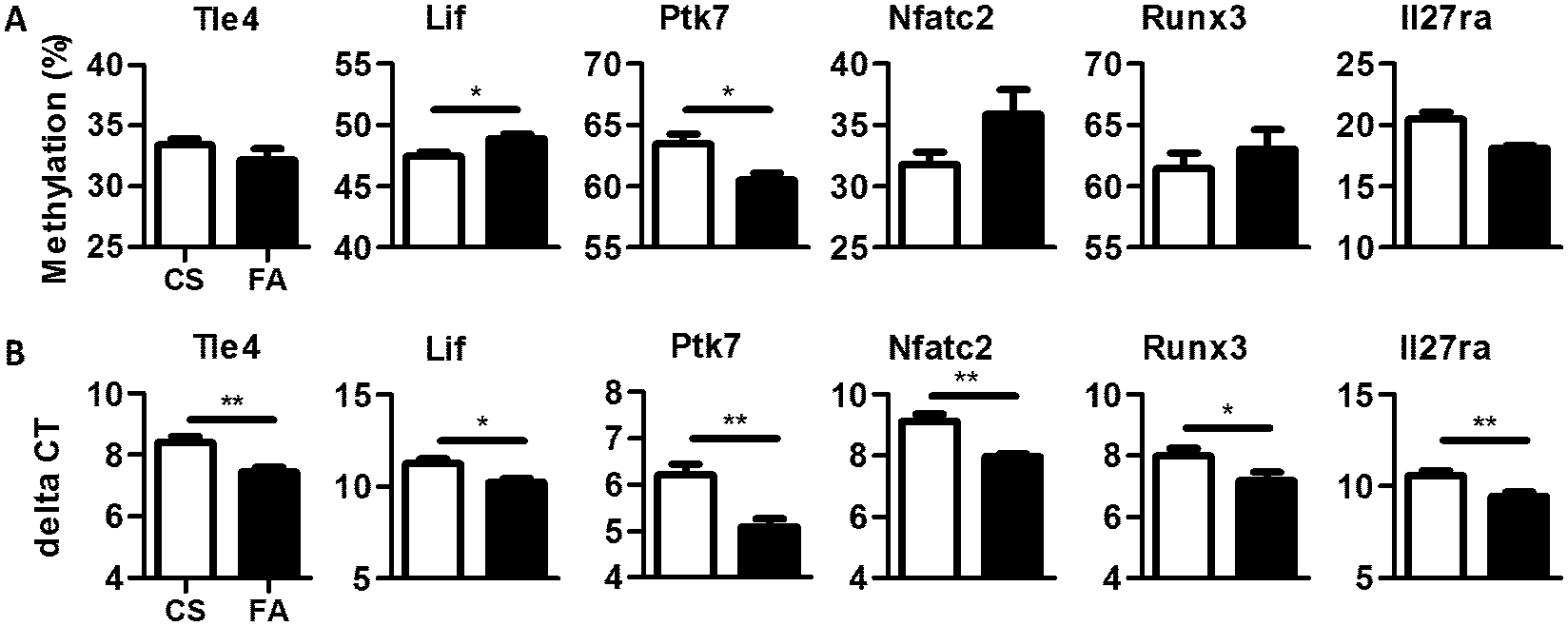
DMR validation is inconsistent despite consistent gene expression differences. (A) Mean methylation levels of 6 selected DMRs for internal validation through pyrosequencing. y-axis = methylation percent (B) Mean transcription levels of 6 selected genes near the 6 selected DMRs. y-axis = delta CT (HDM-treated CS white bar, HDM-treated FA black bar, * p-value < 0.05, ** p-value < 0.01)

DMRs selected for validation from the 99 DMRs (uncorrected p-value < 0.001) were chosen based on known immune function of genes within 25kb of the DMR (Fig 3A and S4 Table). Specific CpGs within each selected DMR were chosen for pyrosequencing based on percent difference between experimental groups, a significant t-test on individual CpGs, and ability to design pyrosequencing primers. DMRs designated Runx3, Tle4, Nfatc2, Lif, Ptk7, and Il27ra were tested. Lif, and Ptk7 validated methyl-sequencing data in addition Nfatc2 had a near significant p-value (0.069) (Fig 3A and S4 Table). It is not unexpected that some of the DMRs did not validate as methyl-sequencing analysis failed to identify any significant targets at genome-wide significance and the reduced threshold increases our type I error.

Transcriptional expression of *Runx3*, *Tle4*, *Nfatc2*, *Lif*, *Ptk7*, and *Il27ra* were measured (Fig 3B). All genes tested demonstrated a significant change in expression with fold-changes ranging from -2.33 to -1.76. Interestingly, the relationship between expression and methylation does not always follow the canonical anti-correlated relationships. The association of gene expression is variable with nearby DMRs suggesting the use of alternative mechanisms in gene regulation.

We also performed subset analyses on methylation clusters within 25kb of asthma related genes as defined by genetic association [25], Ingenuity Pathway Analysis, or overlap of the 2 lists (S5 Table). In aggregate, 329 genes were represented in this analysis. These more targeted analyses focusing on asthma-associated genes did not identify methylation changes associated with *in utero* cigarette smoke exposure. In summary, our results suggest that *in utero* cigarette smoke exposure does not significantly alter the methylome in lung tissue of diseased mice.

## Discussion

Prenatal cigarette exposure is sufficient to alter the severity of HDM-induced allergic airway disease, and these phenotypic changes are associated with specific molecular changes in the lung. However, the association of methylation changes in lung tissue with *in utero* cigarette smoke modified allergic airway disease remains unconvincing. Although methylation changes in the genome-wide and subset analyses were not significant after multiple testing correction, it remains uncertain if smaller shifts in methylation occur as some of the suggestive DMRs were confirmed through pyrosequencing and nearby genes are enriched for immune pathways. In addition, the variable association between methylation and expression suggests that alternative mechanisms may regulate transcriptional activity, and future studies into the epigenetic mechanisms of prenatal cigarette smoke exposure on allergic airway disease should focus on specific cell types and/or alternative mechanisms of transcriptional regulation.

During gestation, the maternal immune system reduces Th1 IFN-γ cell-mediated response to fetal antigens by developing a subtle Th2 state [26]. Fetal immunity reflects that of the mothers, which is dominated by Th2 activity with reduced Th1 function, with underlying epigenetic changes that control gene expression patterns [27]. At birth, the Th1/Th2 cell ratios shift to a proper state; however, continuation of the Th2 state could affect an individual’s risk of developing allergic diseases [28]. Smoking during pregnancy is capable of altering proper immune development leading to reduced innate TLR-mediated response [29], a higher Th2 response and proliferation in cord blood mononuclear cells upon challenge [30, 31], and reduced IFNγ production [32]. The suggestive DMRs in this study highlight immune dysfunction through the enrichment of not just immune pathways in general, but to those important to allergic disease, including IL-4 signaling, NFAT regulation of immune response, CD28 signaling in Th cells, and so on.

A limitation in this study is that the methylation experiment was completed on whole lung tissue with an admixture of cells. Cell type specific expression and methylation patterns increases variance in the analysis creating a higher threshold for discovery. The small phenotypic differences likely could not overcome the noise in the system, and this could explain why DMRs did not reach genome-wide significance. Increased sample size and/or read depth would increase our ability to detect changes and compensate for the small phenotypic changes. The measurement of epigenetic changes in environmental exposure and disease is a powerful tool in studying the etiology of asthma which creates a mechanistic link between environmental exposure and disease phenotype providing additional avenues of research into disease development and severity.

## Supporting Information

S1 Appendix Supplemental Materials and Methods. (DOCX)

S1 Table Agilent SureSelect Methyl Sequencing Summary. (DOCX)

S2 Table Suggestive DMRs in HDM-treated CS vs FA mice. (DOCX)

S3 Table Pathway analysis of Suggestive DMRs in HDM-treated CS and FA mice. (DOCX)

S4 Table DMR Validation and Transcript Levels. (DOCX)

S5 Table DMR Subset Analysis Gene Lists. (DOCX)
