# Supplementary material for: In Utero Cigarette Smoke Affects Allergic Airway Disease But Does Not Alter the Lung Methylome
Source: PLoS One. 2015 Dec 7;10(12):e0144087. doi: 10.1371/journal.pone.0144087 (PMC4671614; doi:10.1371/journal.pone.0144087)
Supplement: S1 Appendix — (DOCX) [file pone.0144087.s001.docx]

**S1 Appendix**

**Supplemental Materials and Methods**

**Mice**

C57BL/6J mice were purchased from Jackson Laboratories. Animals were housed under standard conditions and protocols were approved by the Institutional Animal Care and Use Committee of the University of Colorado Denver.

**Cigarette Smoke Exposure**

12 week old C57BL/6J females were exposed to cigarette smoke at ~50 mg/m^3^ TSP (equivalent to about a pack a day or a heavy smoker) or filtered air for 5 hours/day, 5 days/week for 4 weeks (1 week acclimation included) prior to mating with C57BL/6J males. Cigarette smoke was generated by the TE-10 smoking machine (Teague Enterprises) from 2R4F research cigarettes (University of Kentucky). Cigarette smoke contained a mixture of both side and mainstream smoke. Exposure was continued until birth of pups at which time all exposures were stopped and mothers and pups were placed under normal housing conditions.

House Dust Mite Challenge and Sensitization

Eight- to twelve-week-old C57BL/6^MTHFR-/-^ and C57BL/6 (Charles River) male mice were sensitized to 10 µg house dust mite extract (HDM, GREER Labs) or saline through i.p. injection on days 0 and 7 followed by sensitization on days 14 and 15 with 5 µg HDM or saline administered intratracheally (i.t.) using a microsprayer (Penn Century). Airway function and sample collection occurred on day 17. Prior to administration, HDM was reconstituted in saline to 1 ug/ml and passed through a 0.45 µm filter then a 0.02 µm filter to facilitate microsprayer use.

**Airway physiology**

Mice were anesthetized by an i.p. injection of pentobarbital sodium (60 mg/kg). Following tracheostomy, pancuronium bromide (0.25 mg/kg) was administered, and mice were ventilated on a small animal ventilator (flexiVent FV-FXM1; SCIREQ). Forced oscillation techniques were used to measure respiratory mechanics in response to an aerosolized methacholine challenge (0, 12.5, 25, and 50 mg/ml).

Whole Lung Lavage, Cell Count, and Cell Differential

Whole lung lavage fluid (WLLF) was collected after airway physiology measurement. The lavage fluid was a preparation of 50 mL of 1x PBS with 60 µL of 0.5M EDTA added. Mice were lavaged with an initial 1 mL of lavage fluid followed by two consecutive lavages of 0.5 mL of fresh lavage fluid. WLLF was kept on ice throughout processing.

WLLF was then spun down at 3000 x g for 5 minutes. The supernatant was collected into a separate tube and stored at -80°C. The cell pellet was then treated with 200 µL red blood cell lysing buffer (Sigma) for 1 minute with gentle mixing. 1 mL of cold 1x PBS was immediately added after 1 minute incubation. The cells were then spun down again at 3000 x g for 5 minutes. The supernatant was then discarded and 1 mL of fresh 1x PBS was added to resuspend cells. 20 µL of cell pellet solution was placed in a separate tube and combined with 20 µL Turks solution (10mg Crystal Violet, 3 mL glacial acetic acid, in 100 mL deionized water). 10 µL of solution was placed on a hemacytometer. Cells were counted and total cells were calculated.

Approximately 12,500 cells in 200 µL was placed in a slide funnel assembled with a glass slide and cartridge. The assembly was then placed in cytospin centrifuge (Thermo Scientific Cytospin 4) and run at 700 rpm for 3 minutes. Slide was then removed, placed in a slide rack, and dried overnight. Cells were then stained with Protocol Hema 3 stain set (Fisher), 1 minute solution 1, 3 minutes 45 seconds solution 2, and 1 minute solution 3. After drying, the cover slip was added, and 200 cells were identified and counted based on staining and morphology.

Sample Processing

Blood was withdrawn through cardiac puncture. It was collected in lithium heparin tubes (BD Inc.). Tubes were spun down at 1000 x g for 10 minutes. Serum was then removed and snap frozen in liquid nitrogen and stored at -80°C.

Tissues (spleen, thymus, mediastinal lymph node) collected were snap frozen and stored at -80°C. Lung tissue prior to collection was perfused with 1x PBS. Then, the right lobes were tied off while the left lobe was inflated with a mixture of 50% optimal cutting medium (OCT) and 50% 1x PBS. The inflated left lobe was then quickly removed and embedded in OCT and snap frozen in liquid nitrogen for histology and stored at -80°C. The right lobes were also snap frozen for use in DNA and RNA experiments and stored at -80°C.

ELISA

Sample Preparation

WLLF was concentrated using Amicon Ultra 3K device. 1 mL of WLLF was added to each filter and centrifuged at 4,000 x g for 25 minutes. Then, the filtering device was flipped around and spun for 2 minutes at 1,000 x g. Total volume of the WLLF was concentrated by a factor of 3 on average.

ELISA

IL-4 and IL-5 were measured in the concentrated WLLF and IgE in the serum using ELISA MAX Standard Sets and protocols from BioLegend. First, Costar Assay High Binding 96 well plates were coated with experiment specific capture antibody and incubated at 4°C overnight. Plates were then washed and assay diluent (1% BSA in PBS) was added to each well and incubated at room temperature for 1 hour followed by plate washing. Sample and standard were then added to each well, incubated for 2hours at room temperature then washed. Detection antibody was added to each well, incubated at room temperature for 1 hour, washed, and then treated with Avidin-HRP at room temperature for 30 minutes. Next, the plate underwent two wash cycles prior to the addition of TMB substrate solution. Plates were allowed to develop until desired color was reached, then the reaction was stopped with stop solution. A plate reader was used to measure absorbance at 450 and 570 nm.

Agilent Expression Array

Sample Preparation and Array Processing

A minimum of 25 ng of high quality total RNA from whole lung tissue, as determined by Bioanazlyer RNA assays, was used for SurePrint G3 8x60K arrays (Agilent). One-Color Microarray-Based Gene Expression Analysis: Low Input Quick Amp Labeling protocol version 6.6 and kits were used for this expression experiment. First, RNA was incubated with Spike-in mix, T7 primer, and a cDNA master mix to generate labeled cRNA and purified using RNeasy columns (Qiagen). Then, cRNA was quantified on NanoDrop Spectrometer to determine total concentration and Cyanine 3 dye concentration. 600 ng of labeled sample was then fragmented then hybridized to the arrays for 17 hours followed by washing. Scanning was modified to meet the specifications of the NimbleGen MS200 scanner. A custom parameter on the NimbleGen MS200 scanner was developed in order to scan the Agilent arrays. Images were uploaded to Feature Extraction 11.5.11 software to extract data.

Analysis

Extracted features were up-loaded into Partek Genomics Suite (Partek Inc.), then underwent quantile normalization and log-2 transformation. Analysis of variance (ANOVA) was applied to determine differences between all experimental groups. Adjusted p-values were generated using the FDR q-value selection through Partek.

Bisulfite Sequencing

Sample and Library Preparation

To measure DNA methylation in whole lung tissue, I performed bisulfite sequencing utilizing Agilent’s SureSelect Methyl-Seq Target Enrichment System for Illumina Multiplexed Sequencing. Experimental procedures followed SureSelect Human Methyl-Seq Protocol Version B using SureSelect Methyl-Seq Reagent Kit and SureSelect Mouse Methyl-Seq Capture Library. I started with 4 µg of DNA instead of the recommended 3 µg due to previous experience with similar techniques. Sample DNA was sheared using the Covaris S2 system to fragment the DNA to a 140-180 bp size. Fragmented DNA, then, underwent end repair and purification with AMPure XP beads (Beckman Coulter Genomics). Bioanalyzer DNA 1000 chips were used to confirm the size of DNA fragments. An adenosine was added to the 3’ end of each DNA fragment followed by methylated adaptor ligation. Adaptor-ligated DNA was then purified again using AMPure XP beads then size and quantity was measured through the Bioanalyzer, approximate size of 170-230 bp and minimum of 500 ng. Samples were then dehydrated and reconstituted in 3.4 µL of nuclease-free water to concentrate the sample. Then, samples were hybridized with SureSelect Capture library for approximately 40 hours at 65°C. Immediately after incubation, captured DNA fragments were isolated using Dynabeads MyOne Streptavidin T1 magnetic beads. Captured DNA was then bisulfite converted using Zymo Research’s EZ DNA Methylation-Gold Kit following the SureSelect’s Methyl-Seq modified protocol. Bisulfite-treated libraries were then PCR amplified followed again by AMPure XP bead purification. Libraries were then indexed through PCR amplification. Indexed libraries were purified with AMPure XP beads then assed for quality and quantity with the 2100 Bioanalyzer High Sensitivity DNA assay, size 220-280 bp and a concentration of 2-15 nM. Libraries were pooled to contain either 11 or 12 libraries each at a final DNA concentration of 2 nM per library which was confirmed by a final measurement with the Bioanalyzer. Pooled libraries were then submitted for sequencing on the Illumina HiSeq. Each pool was run twice in separate lanes of a flow cell.

Analysis

Bisulfite-sequencing reads were trimmed and aligned to the mouse genome *mm10* using *bwa-meth* ([16](#_ENREF_16)) which also tabulated percent methylation at each CpG motif. Correlating sets of neighboring CpG sites were clustered together using the Adjacent Site Clustering (A-clustering) algorithm ([17](#_ENREF_17)). Each cluster was required to have a minimum of three CpG sites to constitute a cluster. Clusters were then analyzed using a beta regression weighted based on sequence read depth. Multiple testing correction was performed using the *Benjamini-Hocheberg* method.

Pyrosequencing

DMRs were validated using pyrosequencing. Extracted DNA samples were bisulfite converted using Zymo EZ DNA Methylation-Gold kits and protocol. After conversion and clean-up, converted DNA was measured on a Nanodrop Spectrometer as specified by protocol. 35 ng of converted DNA was then used for the pyrosequencing PCR reaction using Qiagen’s Pyromark PCR kit with each sample run in duplicate. Unmethylated and methylated controls are included for each primer set. Primers for the PCR reaction were designed using the Pyromark Assay Design software. After amplification, the biotinylated-target strand was purified using streptavidin sepharose beads in binding buffer while shaking at room temperature at 1400 rpm for at least 10 minutes. 96 well pyrosequencing plates were then loaded with sequencing primer in annealing buffer and purified target DNA followed by annealing by heating to 80°C for 2 minutes then the plate was allowed to cool to room temperature for 10 minutes. The Pyromark MD was loaded with appropriate volumes of dNTPs, enzyme solution, and substrate solution from the Pyromark Gold Reagents Q96 kit. The plate was then run on the Pyromark MD. Methylation measurements were averaged across duplicates then across experimental group. A one-tailed Mann-Whitney test was used to determine statistical difference between experimental groups.

RT-PCR

Gene expression was measured through quantitative real-time PCR (qRT-PCR) using TaqMan assays (Life Technologies). 80 ng of RNA was converted to cDNA using Superscript III Reverse Transcriptase (Life Technologies) kit and protocol. cDNA was then mixed with TaqMan Fast Advanced Master Mix and best coverage TaqMan assays then run in triplicate on the ViiA 7 Real-Time. Delta CT values were generated from the reference gene, beta-actin. Triplicates were averaged for each sample, and statistical differences were determined through a one-tailed Mann-Whitney test.
