# Supplementary material for: In Utero Cigarette Smoke Affects Allergic Airway Disease But Does Not Alter the Lung Methylome
Source: PLoS One. 2015 Dec 7;10(12):e0144087. doi: 10.1371/journal.pone.0144087 (PMC4671614; doi:10.1371/journal.pone.0144087)
Supplement: S1 Table — (DOCX) [file pone.0144087.s002.docx]

| **S1 Table: Agilent SureSelect Methyl Sequencing Summary** | | | |  |  |  |
| --- | --- | --- | --- | --- | --- | --- |
|  | **n** | **Average Total Reads** | **Mean Coverage** | **2X Coverage (%)** | **20X Coverage (%)** | **Off Target (%)** |
| FA HDM | 9 | 7.2E+07 | 42 | 0.79 | 0.55 | 0.11 |
| FA Saline | 6 | 4.4E+07 | 28 | 0.78 | 0.45 | 0.11 |
| CS HDM | 8 | 6.6E+07 | 40 | 0.79 | 0.52 | 0.11 |
| CS Saline | 6 | 7.2E+07 | 43 | 0.79 | 0.56 | 0.12 |
