# Supplementary material for: In Utero Cigarette Smoke Affects Allergic Airway Disease But Does Not Alter the Lung Methylome
Source: PLoS One. 2015 Dec 7;10(12):e0144087. doi: 10.1371/journal.pone.0144087 (PMC4671614; doi:10.1371/journal.pone.0144087)
Supplement: S2 Table — (DOCX) [file pone.0144087.s003.docx]

| **S2 Table: Suggestive DMRs in HDM-treated CS vs FA mice** | | | | | | |  |  |
| --- | --- | --- | --- | --- | --- | --- | --- | --- |
| **Chrm** | **Start** | **End** | **CpGs** | **coef** | **p-value** | **q-value** | **Gene** | **Distance To Gene** |
| chr4 | 150263029 | 150263062 | 5 | -1.66 | 3.38E-05 | 0.65 | Eno1;Eno1b | 14156;14156 |
| chr4 | 135033681 | 135033743 | 3 | -1.59 | 8.39E-04 | 0.92 | Runx3 | -86901 |
| chr3 | 88042645 | 88042681 | 5 | -1.58 | 9.20E-05 | 0.74 | Gpatch4 | -424 |
| chr11 | 94138540 | 94138670 | 7 | -1.50 | 8.32E-04 | 0.92 | Spag9 | 12458 |
| chr10 | 127092188 | 127092356 | 7 | -1.49 | 7.24E-04 | 0.92 | Agap2 | 0 |
| chr7 | 97325919 | 97325943 | 3 | -1.49 | 4.51E-05 | 0.72 | Usp35 | 0 |
| chr2 | 157901452 | 157901547 | 14 | -1.45 | 6.07E-04 | 0.92 | Ctnnbl1 | 9549 |
| chr5 | 118063299 | 118063307 | 3 | -1.42 | 2.85E-04 | 0.92 | Tesc | 1429 |
| chr18 | 52527547 | 52527593 | 4 | -1.41 | 6.07E-04 | 0.92 | Lox | 0 |
| chr11 | 4247416 | 4247596 | 5 | -1.30 | 5.99E-04 | 0.92 | Osm | 6390 |
| chr16 | 91617026 | 91617046 | 3 | -1.21 | 1.64E-05 | 0.49 | Dnajc28 | 0 |
| chr8 | 105327507 | 105327534 | 3 | -1.21 | 5.48E-04 | 0.92 | Tmem208 | 0 |
| chr4 | 124279006 | 124279019 | 3 | -1.20 | 7.56E-05 | 0.72 | 1700057H15Rik | 170606 |
| chr18 | 61665597 | 61665721 | 5 | -1.11 | 4.63E-04 | 0.92 | Mir143hg | -59 |
| chr5 | 113287153 | 113287386 | 26 | -1.08 | 8.13E-04 | 0.92 | Sgsm1 | 0 |
| chr8 | 85076222 | 85076270 | 4 | -1.05 | 5.00E-04 | 0.92 | Wdr83 | 0 |
| chr19 | 47314049 | 47314161 | 7 | -1.05 | 7.88E-04 | 0.92 | Sh3pxd2a | 0 |
| chr6 | 146852634 | 146852972 | 9 | -1.02 | 4.04E-04 | 0.92 | Smco2 | 0 |
| chr15 | 76542887 | 76542953 | 3 | -1.01 | 7.64E-04 | 0.92 | Slc52a2 | 757 |
| chr2 | 168525888 | 168526023 | 3 | -1.00 | 9.25E-04 | 0.92 | Nfatc2;Nfatc2 | 0;0 |
| chr10 | 63160851 | 63160887 | 4 | -0.97 | 4.01E-04 | 0.92 | Mypn | 0 |
| chr4 | 148734844 | 148734916 | 5 | -0.97 | 8.16E-04 | 0.92 | Gm572 | 63272 |
| chr14 | 7027162 | 7027242 | 9 | -0.94 | 1.85E-05 | 0.49 | Gm10406 | 0 |
| chr18 | 80976446 | 80976498 | 4 | -0.93 | 5.38E-04 | 0.92 | Sall3 | 0 |
| chrX | 159414179 | 159414237 | 4 | -0.93 | 9.09E-04 | 0.92 | Map7d2 | -340 |
| chr11 | 77514877 | 77514912 | 3 | -0.88 | 7.57E-05 | 0.72 | Abhd15 | -204 |
| chr8 | 126476270 | 126476333 | 4 | -0.86 | 2.78E-04 | 0.92 | Gm17296 | -1205 |
| chr10 | 89708838 | 89708877 | 3 | -0.85 | 8.61E-04 | 0.92 | Actr6 | 3095 |
| chr3 | 32396052 | 32396232 | 6 | -0.85 | 9.50E-04 | 0.92 | 4930429B21Rik | 28614 |
| chr5 | 24325380 | 24325485 | 6 | -0.82 | 8.18E-04 | 0.92 | Kcnh2 | 0 |
| chr3 | 123117227 | 123117284 | 3 | -0.81 | 6.87E-05 | 0.72 | Synpo2 | 0 |
| chr2 | 18675994 | 18676001 | 3 | -0.78 | 3.54E-04 | 0.92 | Commd3 | 0 |
| chr13 | 65781856 | 65781921 | 5 | -0.77 | 9.86E-04 | 0.92 | Gm10324 | -331531 |
| chr17 | 43358036 | 43358086 | 5 | -0.72 | 7.21E-04 | 0.92 | Gpr116 | -31379 |
| chr5 | 15936199 | 15936445 | 4 | -0.70 | 6.30E-04 | 0.92 | Cacna2d1 | 0 |
| chr14 | 76109914 | 76110065 | 5 | -0.67 | 7.95E-04 | 0.92 | Gpalpp1 | 0 |
| chr7 | 140955935 | 140955981 | 3 | -0.64 | 2.92E-04 | 0.92 | Ifitm2 | 0 |
| chr8 | 83653791 | 83653980 | 7 | -0.63 | 7.15E-04 | 0.92 | Gipc1 | 0 |
| chr15 | 31368524 | 31368727 | 4 | -0.59 | 4.62E-04 | 0.92 | Ankrd33b | -765 |
| chr16 | 4420943 | 4421145 | 4 | -0.57 | 3.55E-04 | 0.92 | Adcy9 | -445 |
| chr19 | 10773386 | 10773469 | 3 | -0.55 | 7.00E-04 | 0.92 | A430093F15Rik | 0 |
| chr12 | 18515182 | 18515297 | 12 | -0.52 | 6.21E-04 | 0.92 | 5730507C01Rik | 0 |
| chr1 | 89932528 | 89932754 | 12 | -0.50 | 8.83E-04 | 0.92 | Gbx2 | -1352 |
| chr1 | 151087800 | 151087913 | 6 | -0.48 | 5.61E-04 | 0.92 | C730036E19Rik | -50120 |
| chr1 | 64119373 | 64119478 | 5 | -0.38 | 8.48E-04 | 0.92 | Klf7 | 0 |
| chr14 | 8216137 | 8216267 | 4 | 0.62 | 6.85E-05 | 0.72 | Kctd6 | 0 |
| chr6 | 135314332 | 135314366 | 3 | 0.66 | 4.91E-04 | 0.92 | Pbp2 | -3948 |
| chr8 | 127190699 | 127190758 | 3 | 0.68 | 8.70E-04 | 0.92 | Pard3 | 0 |
| chr4 | 127668189 | 127668383 | 4 | 0.75 | 5.40E-04 | 0.92 | CK137956 | 259208 |
| chr3 | 83589324 | 83589379 | 3 | 0.77 | 5.32E-04 | 0.92 | 1700028M03Rik | -15206 |
| chr8 | 122695929 | 122696052 | 4 | 0.80 | 8.53E-04 | 0.92 | Cbfa2t3 | 0 |
| chr14 | 56669126 | 56669214 | 3 | 0.80 | 1.25E-04 | 0.80 | Mphosph8 | 0 |
| chr8 | 25087920 | 25088008 | 4 | 0.82 | 1.01E-03 | 0.92 | Plekha2 | 0 |
| chr9 | 114330005 | 114330112 | 3 | 0.82 | 8.94E-04 | 0.92 | Bcl2a1c | -22 |
| chr12 | 25093649 | 25093707 | 3 | 0.83 | 1.15E-04 | 0.78 | Id2 | 91 |
| chr13 | 65726906 | 65726983 | 5 | 0.85 | 4.27E-04 | 0.92 | Gm10324 | -386469 |
| chr11 | 83648484 | 83648664 | 7 | 0.86 | 6.18E-04 | 0.92 | Ccl3 | 0 |
| chr18 | 38636664 | 38636675 | 3 | 0.87 | 2.63E-04 | 0.92 | Spry4 | -35396 |
| chr18 | 53255464 | 53255528 | 3 | 0.88 | 7.60E-04 | 0.92 | Snx24 | 0 |
| chr4 | 132783608 | 132783640 | 3 | 0.89 | 6.16E-04 | 0.92 | Themis2 | 0 |
| chr3 | 108098501 | 108098798 | 3 | 0.89 | 9.20E-04 | 0.92 | Gnat2 | 0 |
| chr19 | 56344247 | 56344259 | 3 | 0.89 | 7.68E-04 | 0.92 | Nrap | 0 |
| chr6 | 86496987 | 86497069 | 3 | 1.00 | 7.55E-05 | 0.72 | 2310040G24Rik | -8760 |
| chr11 | 119937292 | 119937414 | 5 | 1.00 | 1.17E-04 | 0.78 | Baiap2 | -5677 |
| chr18 | 73835755 | 73835925 | 3 | 1.02 | 4.28E-04 | 0.92 | Me2 | -20363 |
| chr4 | 139974780 | 139974826 | 3 | 1.02 | 4.81E-04 | 0.92 | Klhdc7a | -6754 |
| chr11 | 62996643 | 62996682 | 3 | 1.09 | 6.35E-04 | 0.92 | Mir6354 | 0 |
| chr2 | 61811483 | 61811544 | 3 | 1.09 | 8.28E-04 | 0.92 | Tbr1 | 0 |
| chr11 | 84522053 | 84522086 | 3 | 1.10 | 6.33E-04 | 0.92 | Lhx1 | 0 |
| chr8 | 84063944 | 84064070 | 4 | 1.10 | 3.71E-04 | 0.92 | Rfx1 | -2765 |
| chr7 | 66387811 | 66387889 | 8 | 1.10 | 9.38E-04 | 0.92 | Lrrk1 | 0 |
| chr5 | 134729315 | 134729388 | 3 | 1.12 | 2.47E-04 | 0.92 | Eln | 0 |
| chr15 | 34016791 | 34016973 | 4 | 1.13 | 3.11E-05 | 0.65 | Mtdh | -65745 |
| chr17 | 46552275 | 46552434 | 5 | 1.18 | 9.65E-04 | 0.92 | Srf | 0 |
| chr5 | 36868773 | 36868787 | 4 | 1.23 | 2.04E-04 | 0.92 | Ppp2r2c | 0 |
| chr17 | 73037161 | 73037270 | 3 | 1.25 | 5.01E-04 | 0.92 | Lclat1 | -70714 |
| chr4 | 95106226 | 95106315 | 3 | 1.25 | 9.40E-05 | 0.74 | Jun | -54004 |
| chr11 | 49719835 | 49719888 | 3 | 1.29 | 7.83E-04 | 0.92 | Cnot6 | -7113 |
| chr1 | 156615798 | 156616051 | 4 | 1.29 | 7.18E-05 | 0.72 | Abl2 | 0 |
| chr11 | 95046878 | 95047005 | 3 | 1.31 | 8.27E-05 | 0.74 | Itga3 | 0 |
| chr6 | 113379994 | 113380143 | 3 | 1.36 | 8.68E-04 | 0.92 | Arpc4;Arpc4 | 0;0 |
| chr12 | 109542822 | 109542870 | 3 | 1.38 | 6.78E-04 | 0.92 | Meg3 | 0 |
| chr9 | 21125858 | 21125923 | 3 | 1.39 | 5.76E-04 | 0.92 | Tyk2 | 0 |
| chr14 | 122373730 | 122373779 | 4 | 1.42 | 7.07E-04 | 0.92 | Clybl;Gm5089 | 0;0 |
| chr15 | 101277572 | 101277651 | 5 | 1.42 | 9.48E-04 | 0.92 | Nr4a1 | 2778 |
| chr2 | 118728223 | 118728285 | 3 | 1.46 | 5.46E-06 | 0.25 | Plcb2 | 0 |
| chr8 | 105835357 | 105835381 | 3 | 1.48 | 1.09E-04 | 0.78 | Tsnaxip1 | 0 |
| chr11 | 32552724 | 32552917 | 3 | 1.49 | 3.35E-04 | 0.92 | Stk10 | 0 |
| chr10 | 46235841 | 46236012 | 3 | 1.55 | 7.39E-04 | 0.92 | Snord14d | -8735 |
| chr10 | 7867202 | 7867307 | 4 | 1.56 | 6.85E-04 | 0.92 | Zc3h12d | 0 |
| chr2 | 165332955 | 165333024 | 5 | 1.59 | 3.52E-04 | 0.92 | Elmo2 | -6476 |
| chr11 | 51644368 | 51644441 | 7 | 1.60 | 3.20E-04 | 0.92 | N4bp3 | 0 |
| chr8 | 121951636 | 121951670 | 3 | 1.62 | 2.17E-04 | 0.92 | Banp | 0 |
| chr2 | 33219624 | 33219647 | 3 | 1.66 | 7.43E-04 | 0.92 | Angptl2;Ralgps1 | 0;0 |
| chr3 | 103737837 | 103737888 | 3 | 1.69 | 1.75E-06 | 0.23 | Olfml3;Mir3473f | 0;0 |
| chr11 | 77498744 | 77498775 | 3 | 1.80 | 6.40E-04 | 0.92 | Git1 | 0 |
| chr11 | 105181678 | 105181764 | 8 | 2.08 | 8.66E-04 | 0.92 | Tlk2 | 0 |
| chr1 | 143777067 | 143777101 | 3 | 2.38 | 4.03E-04 | 0.92 | Trove2 | -16 |
| chr9 | 46012706 | 46012730 | 3 | 2.62 | 2.05E-04 | 0.92 | Sik3 | -89 |
| chr19 | 24280501 | 24280538 | 5 | 3.35 | 5.60E-06 | 0.25 | Fxn | 0 |
