# Supplementary material for: In Utero Cigarette Smoke Affects Allergic Airway Disease But Does Not Alter the Lung Methylome
Source: PLoS One. 2015 Dec 7;10(12):e0144087. doi: 10.1371/journal.pone.0144087 (PMC4671614; doi:10.1371/journal.pone.0144087)
Supplement: S3 Table — (DOCX) [file pone.0144087.s004.docx]

| **S3 Table: Pathway analysis of Suggestive DMRs in HDM-treated CS and FA mice** | | |  |
| --- | --- | --- | --- |
| **Ingenuity Canonical Pathways** | **-log(p-value)** | **Ratio** | **Molecules** |
| Rac Signaling | 4.08 | 0.05 | ITGA3,JUN,BAIAP2,PARD3,ARPC4 |
| PDGF Signaling | 3.48 | 0.05 | JUN,ABL2,TYK2,SRF |
| CXCR4 Signaling | 3.31 | 0.03 | ADCY9,PLCB2,JUN,GNAT2,ELMO2 |
| Signaling by Rho Family GTPases | 3.30 | 0.03 | ITGA3,JUN,BAIAP2,GNAT2,PARD3,ARPC4 |
| Cdc42 Signaling | 3.12 | 0.03 | ITGA3,JUN,BAIAP2,PARD3,ARPC4 |
| Actin Nucleation by ARP-WASP Complex | 2.75 | 0.05 | ITGA3,BAIAP2,ARPC4 |
| PI3K Signaling in B Lymphocytes | 2.65 | 0.03 | PLCB2,JUN,NFATC2,PLEKHA2 |
| Axonal Guidance Signaling | 2.60 | 0.02 | PLCB2,ITGA3,BAIAP2,GNAT2,NFATC2,GIT1,ARPC4 |
| Cardiac Hypertrophy Signaling | 2.57 | 0.02 | ADCY9,PLCB2,JUN,SRF,GNAT2 |
| Molecular Mechanisms of Cancer | 2.32 | 0.02 | ADCY9,PLCB2,ITGA3,JUN,TYK2,GNAT2 |
| Role of JAK family kinases in IL-6-type Cytokine Signaling | 2.29 | 0.08 | TYK2,OSM |
| Gluconeogenesis I | 2.29 | 0.08 | ENO1,ME2 |
| Role of NFAT in Regulation of the Immune Response | 2.20 | 0.02 | PLCB2,JUN,GNAT2,NFATC2 |
| Endothelin-1 Signaling | 2.20 | 0.02 | ADCY9,PLCB2,JUN,GNAT2 |
| Regulation of Actin-based Motility by Rho | 2.15 | 0.03 | ITGA3,BAIAP2,ARPC4 |
| IL-1 Signaling | 2.15 | 0.03 | ADCY9,JUN,GNAT2 |
| CDK5 Signaling | 2.05 | 0.03 | ADCY9,ITGA3,PPP2R2C |
| Oncostatin M Signaling | 2.03 | 0.06 | TYK2,OSM |
| Cholecystokinin/Gastrin-mediated Signaling | 2.03 | 0.03 | PLCB2,JUN,SRF |
| Interferon Signaling | 1.98 | 0.06 | TYK2,IFITM2 |
| fMLP Signaling in Neutrophils | 1.95 | 0.03 | PLCB2,NFATC2,ARPC4 |
| April Mediated Signaling | 1.94 | 0.05 | JUN,NFATC2 |
| Corticotropin Releasing Hormone Signaling | 1.91 | 0.03 | ADCY9,JUN,NR4A1 |
| B Cell Activating Factor Signaling | 1.90 | 0.05 | JUN,NFATC2 |
| Actin Cytoskeleton Signaling | 1.85 | 0.02 | ITGA3,BAIAP2,GIT1,ARPC4 |
| CD28 Signaling in T Helper Cells | 1.85 | 0.03 | JUN,NFATC2,ARPC4 |
| P2Y Purigenic Receptor Signaling Pathway | 1.84 | 0.03 | ADCY9,PLCB2,JUN |
| iNOS Signaling | 1.82 | 0.05 | JUN,TYK2 |
| RhoA Signaling | 1.81 | 0.02 | ABL2,BAIAP2,ARPC4 |
| PI3K/AKT Signaling | 1.80 | 0.02 | ITGA3,TYK2,PPP2R2C |
| Cellular Effects of Sildenafil (Viagra) | 1.75 | 0.02 | ADCY9,PLCB2,KCNH2 |
| GNRH Signaling | 1.75 | 0.02 | ADCY9,PLCB2,JUN |
| Phospholipase C Signaling | 1.71 | 0.02 | ADCY9,PLCB2,ITGA3,NFATC2 |
| Relaxin Signaling | 1.69 | 0.02 | ADCY9,JUN,GNAT2 |
| Synaptic Long Term Depression | 1.64 | 0.02 | PLCB2,GNAT2,PPP2R2C |
| Role of CHK Proteins in Cell Cycle Checkpoint Control | 1.63 | 0.04 | PPP2R2C,TLK2 |
| EGF Signaling | 1.62 | 0.04 | JUN,SRF |
| Epithelial Adherens Junction Signaling | 1.61 | 0.02 | BAIAP2,PARD3,ARPC4 |
| ErbB2-ErbB3 Signaling | 1.61 | 0.04 | JUN,TYK2 |
| ATM Signaling | 1.58 | 0.03 | JUN,TLK2 |
| Tec Kinase Signaling | 1.52 | 0.02 | ITGA3,TYK2,GNAT2 |
| Calcium-induced T Lymphocyte Apoptosis | 1.51 | 0.03 | NR4A1,NFATC2 |
| Dopamine-DARPP32 Feedback in cAMP Signaling | 1.50 | 0.02 | ADCY9,PLCB2,PPP2R2C |
| Hepatic Cholestasis | 1.49 | 0.02 | ADCY9,JUN,OSM |
| GABA Receptor Signaling | 1.48 | 0.03 | ADCY9,KCNH2 |
| IL-10 Signaling | 1.46 | 0.03 | JUN,TYK2 |
| Agrin Interactions at Neuromuscular Junction | 1.45 | 0.03 | ITGA3,JUN |
| CREB Signaling in Neurons | 1.43 | 0.02 | ADCY9,PLCB2,GNAT2 |
| Chemokine Signaling | 1.43 | 0.03 | PLCB2,JUN |
| JAK/Stat Signaling | 1.42 | 0.03 | JUN,TYK2 |
| RhoGDI Signaling | 1.42 | 0.02 | ITGA3,GNAT2,ARPC4 |
| Ephrin Receptor Signaling | 1.41 | 0.02 | ITGA3,GNAT2,ARPC4 |
| Role of Macrophages, Fibroblasts and Endothelial Cells in Rheumatoid Arthritis | 1.41 | 0.01 | PLCB2,JUN,NFATC2,OSM |
| LPS-stimulated MAPK Signaling | 1.41 | 0.03 | JUN,SRF |
| Leptin Signaling in Obesity | 1.40 | 0.03 | ADCY9,PLCB2 |
| PPARÎ±/RXRÎ± Activation | 1.38 | 0.02 | ADCY9,PLCB2,JUN |
| IL-4 Signaling | 1.38 | 0.03 | TYK2,NFATC2 |
| Production of Nitric Oxide and Reactive Oxygen Species in Macrophages | 1.38 | 0.02 | JUN,TYK2,PPP2R2C |
| Dopamine Receptor Signaling | 1.36 | 0.03 | ADCY9,PPP2R2C |
| Regulation of the Epithelial-Mesenchymal Transition Pathway | 1.35 | 0.02 | LOX,ID2,TYK2 |
| Regulation of IL-2 Expression in Activated and Anergic T Lymphocytes | 1.35 | 0.03 | JUN,NFATC2 |
| Ceramide Signaling | 1.34 | 0.03 | JUN,PPP2R2C |
| ERK/MAPK Signaling | 1.34 | 0.02 | ITGA3,SRF,PPP2R2C |
| Thrombin Signaling | 1.31 | 0.02 | ADCY9,PLCB2,GNAT2 |
| Breast Cancer Regulation by Stathmin1 | 1.31 | 0.02 | ADCY9,PLCB2,PPP2R2C |
