# Supplementary material for: In Utero Cigarette Smoke Affects Allergic Airway Disease But Does Not Alter the Lung Methylome
Source: PLoS One. 2015 Dec 7;10(12):e0144087. doi: 10.1371/journal.pone.0144087 (PMC4671614; doi:10.1371/journal.pone.0144087)
Supplement: S4 Table — (DOCX) [file pone.0144087.s005.docx]

| **S4 Table: DMR Validation and Transcript Levels** | | | | |  |  |  |  |  |
| --- | --- | --- | --- | --- | --- | --- | --- | --- | --- |
|  |  | **Methyl-Sequencing Data** | | |  | **Pyrosequencing Data** | | |  |
| **Chrm** | **postion** | **HDM-FA** | **HDM-CS** | **Difference** | **DMR p-value** | **HDM-FA** | **HDM-CS** | **Difference** | **p-value** |
| chr11 | 4247417 | 0.75 | 0.47 | -0.28 | 6.0E-04 | 0.49 | 0.47 | -0.02 | **0.014** |
| chr17 | 46552350 | 0.48 | 0.82 | 0.34 | 9.6E-04 | 0.61 | 0.63 | 0.02 | **0.006** |
| chr19 | 14890412 | 0.15 | 0.25 | 0.10 | 1.1E-03 | 0.22 | 0.23 | 0.01 | 0.139 |
| chr2 | 168525953 | 0.61 | 0.42 | -0.19 | 9.2E-04 | 0.36 | 0.32 | -0.04 | 0.069 |
| chr4 | 135033743 | 0.83 | 0.48 | -0.35 | 8.4E-04 | 0.62 | 0.60 | -0.02 | 0.228 |
| chr8 | 84064048 | 0.11 | 0.25 | 0.14 | 3.7E-04 | 0.19 | 0.20 | 0.01 | 0.109 |
