# Supplementary material for: In Utero Cigarette Smoke Affects Allergic Airway Disease But Does Not Alter the Lung Methylome
Source: PLoS One. 2015 Dec 7;10(12):e0144087. doi: 10.1371/journal.pone.0144087 (PMC4671614; doi:10.1371/journal.pone.0144087)
Supplement: S5 Table — (DOCX) [file pone.0144087.s006.docx]

| **S5 Table: DMR Subset Analysis Gene Lists** | | |  | |  |
| --- | --- | --- | --- | --- | --- |
| Genetic Association | IPA Asthma Associated |  | | Genetic Association | IPA Asthma Associated |
| 1700012D01Rik | 1700012D01Rik |  | | Kcnh2 |  |
| 1700119H24Rik | 1700119H24Rik |  | | Kcnh6 |  |
| 4930455D15Rik | 4930455D15Rik |  | | Kcnn1 |  |
| 4930488B22Rik |  |  | | **Kdsr** | **Kdsr** |
| 9130409I23Rik |  |  | | Kif3a | Kif3a |
| 9830166K06Rik | 9830166K06Rik |  | | Kmo |  |
| Abcb8 |  |  | | **Lep** | **Lep** |
| Ace |  |  | | Lepr |  |
| Ace3 |  |  | | Lgals9 | Lgals9 |
| Actl7b |  |  | | Lifr |  |
| Actr8 | Actr8 |  | | Llph | Llph |
| Adam18 |  |  | | Lrp1 | Lrp1 |
| Adam33 |  |  | | **Lst1** | **Lst1** |
| Adamts12 |  |  | | **Lta** | **Lta** |
| Adh4 | Adh4 |  | | Lta4h |  |
| Adh5 | Adh5 |  | | **Ltb** | **Ltb** |
| Adora1 | Adora1 |  | | Ltc4s | Ltc4s |
| Adora2a | Adora2a |  | | Ly86 |  |
| Adra1b | Adra1b |  | | Maml1 | Maml1 |
| Adrb2 | Adrb2 |  | | Mast3 |  |
| Agt |  |  | | Mbl2 |  |
| Ahi1 |  |  | | Med23 | Med23 |
| Ahnak |  |  | | Med7 | Med7 |
| Aicda |  |  | | Mefv |  |
| **Alas1** | **Alas1** |  | | Mfap5 |  |
| Alox5ap |  |  | | Mfsd12 | Mfsd12 |
| Aoah |  |  | | Mgat4b | Mgat4b |
| Ap1s1 |  |  | | Mif |  |
| Apobec1 |  |  | | **Mir3098** | **Mir3098** |
| Apof |  |  | | Mir3470b |  |
| Apon |  |  | | Mir5098 |  |
| Arg1 | Arg1 |  | | Mir6989 | Mir6989 |
| Arg2 | Arg2 |  | | Mir7240 |  |
| Arrdc2 |  |  | | Mir760 |  |
| Atg9b |  |  | | Mir7671 |  |
| **B9d2** | **B9d2** |  | | Mir7688 |  |
| BC021614 |  |  | | **Mmp9** | **Mmp9** |
| **Bcl2** | **Bcl2** |  | | **Mpdu1** | **Mpdu1** |
| Bco2 | Bco2 |  | | Mpo | Mpo |
| Bdnf |  |  | | Mrc1 |  |
| Blmh |  |  | | **Mrpl4** | **Mrpl4** |
| Bzrap1 | Bzrap1 |  | | Ms4a2 | Ms4a2 |
| **C3** | **C3** |  | | Mtmr12 |  |
| Cabin1 |  |  | | Muc2 |  |
| Cactin | Cactin |  | | Muc5ac |  |
| Cadm4 |  |  | | Mvd |  |
| Calm3 |  |  | | Myb |  |
| Capn9 |  |  | | Mybph | Mybph |
| Cat |  |  | | Myl4 |  |
| **Ccdc97** | **Ccdc97** |  | | Mylk | Mylk |
| Ccl24 | Ccl24 |  | | Nab2 | Nab2 |
| **Ccl7** | **Ccl7** |  | | Nat1 |  |
| **Ccr3** | **Ccr3** |  | | Nat2 |  |
| **Cd68** | **Cd68** |  | | Nat3 |  |
| Cd86 |  |  | | Ndufa2 |  |
| **Cflar** | **Cflar** |  | | Ndufv1 |  |
| Cftr |  |  | | **Necap1** | **Necap1** |
| Chdh | Chdh |  | | **Nfkbil1** | **Nfkbil1** |
| Chil1 | Chil1 |  | | Nob1 |  |
| Ckap2l |  |  | | **Nod1** | **Nod1** |
| Clec16a |  |  | | Nos1 |  |
| **Csf2** | **Csf2** |  | | Nos2 | Nos2 |
| Ctla4 |  |  | | Nos3 |  |
| Ctnna3 | Ctnna3 |  | | Npsr1 | Npsr1 |
| Cttn |  |  | | Nqo1 |  |
| Cxcl12 |  |  | | Opn3 |  |
| Cyb561 |  |  | | Ormdl3 | Ormdl3 |
| Cyba |  |  | | Osbpl7 | Osbpl7 |
| Cyfip2 |  |  | | Pan2 |  |
| Cyp1b1 |  |  | | Parp1 |  |
| Cyp24a1 |  |  | | Pde4d | Pde4d |
| Cyp2r1 | Cyp2r1 |  | | Pdgfra |  |
| Cysltr1 | Cysltr1 |  | | Pfdn4 |  |
| Dact3 |  |  | | Pip5k1c | Pip5k1c |
| Dap3 |  |  | | Plau |  |
| Ddt |  |  | | Plaur |  |
| Defb1 |  |  | | Pon1 |  |
| Dennd1b |  |  | | **Pparg** | **Pparg** |
| Derl3 |  |  | | **Ppm1m** | **Ppm1m** |
| Dnajc12 | Dnajc12 |  | | Ppp1r9a |  |
| Dnttip2 |  |  | | Prlr |  |
| Dpp10 |  |  | | Prm1 |  |
| E130310I04Rik | E130310I04Rik |  | | Prn |  |
| Edn1 |  |  | | Prnd |  |
| Egflam |  |  | | Prnp |  |
| **Eif4a1** | **Eif4a1** |  | | Psmb8 |  |
| Elf5 |  |  | | Psmb9 |  |
| Ephx1 |  |  | | Ptgdr | Ptgdr |
| **Ezh2** | **Ezh2** |  | | Ptger2 | Ptger2 |
| F3 |  |  | | Ptger3 |  |
| Fam114a1 |  |  | | Ptger4 |  |
| Fam206a |  |  | | Ptgir |  |
| Fbxo21 |  |  | | Ptgs2 | Ptgs2 |
| **Foxj2** | **Foxj2** |  | | Ptprd |  |
| Fyn |  |  | | Ptpre |  |
| Gclm |  |  | | Rad50 | Rad50 |
| Gfra4 |  |  | | Rara |  |
| **Ggct** | **Ggct** |  | | Rdh11 | Rdh11 |
| Gipc3 | Gipc3 |  | | Rhbdf1 | Rhbdf1 |
| Gjd3 |  |  | | Rnu6 |  |
| Gm11651 |  |  | | Rrp15 |  |
| Gm12 | Gm12 |  | | Scgb1a1 |  |
| Gm12216 |  |  | | Sdhd | Sdhd |
| Gm14023 |  |  | | **Sell** | **Sell** |
| Gm16894 |  |  | | Serpine1 |  |
| Gm19583 |  |  | | **Slc12a5** | **Slc12a5** |
| Gng8 |  |  | | Slc25a48 | Slc25a48 |
| **Gpr108** | **Gpr108** |  | | Slc3a2 | Slc3a2 |
| Gpx1 |  |  | | Slc6a4 |  |
| Grb7 | Grb7 |  | | Socs1 |  |
| Gstp2 |  |  | | Sod2 |  |
| Gstt1 |  |  | | Specc1l | Specc1l |
| Gstt2 |  |  | | Sqstm1 | Sqstm1 |
| Gstt3 |  |  | | Stard5 | Stard5 |
| Gstt4 |  |  | | Stat1 |  |
| Hacd2 | Hacd2 |  | | Stat2 |  |
| Hal |  |  | | Stat4 |  |
| Hdac7 | Hdac7 |  | | Stat6 | Stat6 |
| Hip1 |  |  | | Syn1 |  |
| Hlx |  |  | | Tap1 |  |
| Hmg20b | Hmg20b |  | | Tap2 |  |
| Hmox1 | Hmox1 |  | | Tbkbp1 | Tbkbp1 |
| Hnmt | Hnmt |  | | Tbx21 | Tbx21 |
| **Icam1** | **Icam1** |  | | Tbxa2r | Tbxa2r |
| **Icam4** | **Icam4** |  | | Tex12 | Tex12 |
| **Icam5** | **Icam5** |  | | **Tgfb1** | **Tgfb1** |
| Ido1 |  |  | | Tgfb2 |  |
| Ido2 |  |  | | Timd4 |  |
| Ifna1 |  |  | | Timeless |  |
| Ifnar1 |  |  | | Tle4 |  |
| Ifne |  |  | | **Tlr2** | **Tlr2** |
| **Ifng** | **Ifng** |  | | Tlr6 |  |
| Ifngr2 |  |  | | Tlr8 |  |
| Igfbp4 |  |  | | **Tlr9** | **Tlr9** |
| Ikbkap |  |  | | Tmbim4 | Tmbim4 |
| Ikzf3 | Ikzf3 |  | | Tmco6 |  |
| **Il10** | **Il10** |  | | Tmem194 | Tmem194 |
| Il12a |  |  | | Tmem50b |  |
| Il12b | Il12b |  | | Tmem63a |  |
| Il12rb1 |  |  | | **Tmem91** | **Tmem91** |
| Il13 | Il13 |  | | Tnc |  |
| Il16 | Il16 |  | | **Tnf** | **Tnf** |
| Il17c |  |  | | **Tnfsf13** | **Tnfsf13** |
| Il17rb | Il17rb |  | | **Tnfsf14** | **Tnfsf14** |
| Il18r1 |  |  | | **Tnfsfm13** | **Tnfsfm13** |
| Il1a |  |  | | Tnp2 |  |
| Il1r2 |  |  | | Tom1 | Tom1 |
| Il1rl1 | Il1rl1 |  | | Top2a |  |
| Il1rn | Il1rn |  | | Traf3ip2 |  |
| Il21 |  |  | | **Trip10** | **Trip10** |
| Il23a |  |  | | Trnt1 |  |
| **Il3** | **Il3** |  | | Trpd52l3 | Trpd52l3 |
| Il33 | Il33 |  | | Ttc1 | Ttc1 |
| Il4 | Il4 |  | | **Twf2** | **Twf2** |
| Il5 | Il5 |  | | **Tyms** | **Tyms** |
| Il5ra |  |  | | **Vcam1** | **Vcam1** |
| **Il6** | **Il6** |  | | Vdr | Vdr |
| Il9r | Il9r |  | | Vti1b | Vti1b |
| Inpp4a |  |  | | Wdr36 | Wdr36 |
| Irak1 |  |  | | **Wnt5a** | **Wnt5a** |
| Irak3 | Irak3 |  | | Wtap |  |
| Irf1 |  |  | | Zfp263 |  |
| Irgc1 |  |  | | Zfr |  |
| **Itga4** | **Itga4** |  | | Zpbp2 | Zpbp2 |
| Itgb3 |  |  | |  |  |
| **bold = overlap** |  |  | |  |  |
